# Heterologous prime-boost with AdC68- and mRNA-based COVID-19 vaccines elicit potent immune responses in mice

**DOI:** 10.1038/s41392-021-00843-6

**Published:** 2021-12-13

**Authors:** Wenjuan Li, Xingxing Li, Danhua Zhao, Jingjing Liu, Ling Wang, Miao Li, Xinyu Liu, Jia Li, Xiaohong Wu, Yuhua Li

**Affiliations:** Department of Arboviral Vaccine, National Institutes for Food and Drug Control, 102629 Beijing, China

**Keywords:** Vaccines, Vaccines

**Dear Editor**

An optimal vaccination program that can elicit long-lasting humoral and cellular immune response is key to long-term prevention of coronavirus disease 2019 (COVID-19). Multiple variants of severe acute respiratory syndrome coronavirus 2 (SARS-CoV-2) continue to emerge. The neutralizing capacities of available COVID-19 vaccines against these variants have declined.^1^ In such a scenario, heterologous prime-boost programs for COVID-19 vaccines have attracted considerable interest. A clinical trial showed that heterologous prime-boost vaccination of ChAdOx1 nCoV-19 and BNT162b2 produced strong humoral and cellular immune responses, and high titers of neutralizing antibodies against the B.1.1.7, B.1.351, and P.1 variants of SARS-CoV-2 were observed.^2^

We evaluated different prime-boost strategies with a chimpanzee adenovirus vector AdC68-based vaccine ChAdTS-S,^3^ and an mRNA-based vaccine ARCoV,^4^ in four-week-old female BALB/c mice. The prime-boost designs are outlined in Fig. 1a. ChAdTS-S was administered either intranasally (i.n.) or intramuscularly (i.m.), and ARCoV was administered through the intramuscular (i.m.) route. To the best of our knowledge, our effort is a novel study to evaluate heterologous prime-boost strategies of COVID-19 vaccines using different vaccination routes.

**Fig. 1:**
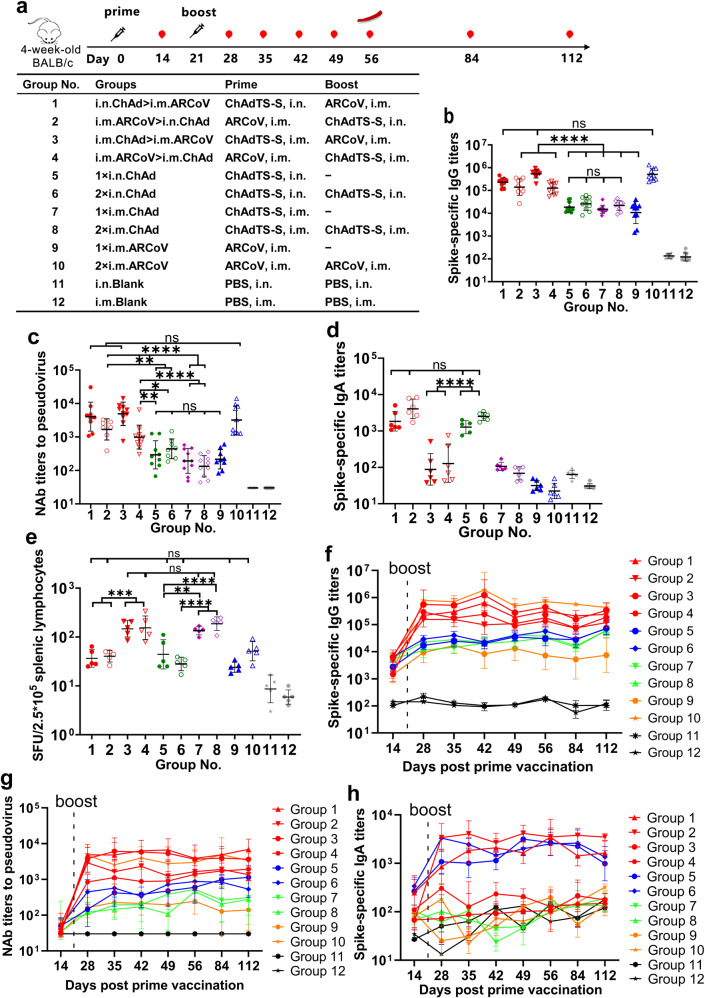
Design and characteristics of potent immune response of heterologous prime-boost with ChAdTS-S and ARCoV. **a** Overall scheme of the group design, vaccination, and immunological characterization.  indicates vaccination, 5 × 10^9^ VP of ChAdTS-S or 6 μg of ARCoV were used for each vaccination.  indicates blood sample collection.  indicates splenic lymphocyte isolation. i.n. indicates intranasal vaccination. i.m. indicates intramuscular vaccination. − indicates no boost vaccination. **b**–**d** The serum spike-specific binding IgG titers (**b**), pseudovirus NAb titers (**c**), and spike-specific binding IgA titers (**d**) on day 35 after prime immunization (*N* = 7–10 per group). **e** T cell response to SARS-CoV-2 spike peptides measured using an IFN-γ ELISpot on day 56 after the prime immunization (*N* = 5 per group). **f**–**h** Temporal changes in the serum spike-specific IgG titers (**f**), pseudovirus NAb titers (**g**), and spike-specific IgA titers (**h**) for up to 112 days after the prime immunization (*N* = 5–6 per group). In the VSV-based pseudovirus assay, NAb titers less than 30 were recorded as 30 when plotting the figures. Bars represent the geometric means with geometric SDs, *p < 0.05; **p < 0.01; ***p < 0.001; ****p < 0.0001; ns: *p* > 0.05. SFU spot forming unit

The spike-specific IgG titers in serum were detected using ELISA on day 35 after prime immunization to evaluate the systemic immune responses (Fig. 1b). The heterologous ChAdTS-S prime and ARCoV boost (groups i.n.ChAd**>**i.m.ARCoV and i.m.ChAd**>**i.m.ARCoV) or two doses of ARCoV (group 2×i.m.ARCoV) induced the strongest spike-specific IgG responses among all the tested groups. The spike-specific IgG geometric mean titers (GMTs) of the groups inoculated with heterologous ARCoV prime and ChAdTS-S boost (groups i.m.ARCoV**>**i.n.ChAd and i.m.ARCoV**>**i.m.ChAd) were higher than those induced in groups 1×i.n.ChAd, 2×i.n.ChAd, 1×i.m.ChAd, 2×i.m.ChAd, and 1×i.m.ARCoV. One dose of intranasal ChAdTS-S immunization (group 1×i.n.ChAd) induced spike-specific IgG responses as strong as that induced by one dose of intramuscular ChAdTS-S immunization (group 1×i.m.ChAd). Homologous ChAdTS-S boost via either the intranasal or intramuscular route did not significantly elevate the spike-specific IgG responses. Groups 1×i.n.ChAd, 2×i.n.ChAd, 1×i.m.ChAd, and 2×i.m.ChAd had no significant differences with each other.

The neutralization ability in the serum was assessed using a vesicular stomatitis virus (VSV)-based pseudovirus assay^5^ at day 35 after prime immunization (Fig. 1c). The neutralizing antibody (NAb) titers of heterologous ChAdTS-S prime (i.n. or i.m.) and ARCoV boost (groups i.n.ChAd**>**i.m.ARCoV and i.m.ChAd**>**i.m.ARCoV) or two doses of ARCoV (group 2×i.m.ARCoV) were the highest among all the tested groups, with no significant differences among these three groups. Groups administered the heterologous ARCoV prime and ChAdTS-S boost (i.n. or i.m., groups i.m.ARCoV**>**i.n.ChAd and i.m.ARCoV**>**i.m.ChAd) had stronger neutralizing responses than groups 1×i.n.ChAd, 2×i.n.ChAd, 1×i.m.ChAd, 2×i.m.ChAd, and 1×i.m.ARCoV. Homologous ChAdTS-S boost (i.n. or i.m.) did not significantly elevate the neutralizing response. The NAb GMTs of i.n.ChAdTS-S immunization groups (422 for group 1×i.n.ChAd and 571 for group 2×i.n.ChAd) were higher than those of i.m.ChAdTS-S immunization groups (193 for group 1×i.m.ChAdT and 146 for group 2×i.m.ChAd). However, there were no significant statistical differences among these four groups.

The mucosal immunity level was assessed by detecting the spike-specific IgA titers in the serum using ELISA on day 35 after the prime immunization (Fig. 1d). All the groups administered at least one dose of i.n.ChAd (i.n.ChAd>i.m.ARCoV, i.m.ARCoV>i.n.ChAd, 1×i.n.ChAd, and 2×i.n.ChAd) exhibited high spike-specific IgA titers with no significant differences, indicating high mucosal immunity levels. Groups i.m.ARCoV>i.n.ChAd and 2×i.n.ChAd had the highest IgA GMTs. The second dose of i.n.ChAdTS-S boost did not significantly elevate the mucosal immunity level. The other groups administered without i.n.ChAd (i.m.ChAd>i.m.ARCoV, i.m.ARCoV>i.m.ChAd, 1×i.m.ChAd, 2×i.m.ChAd, 1×i.m.ARCoV, 2×i.m.ARCoV, i.n.Blank, and i.m.Blank) did not elicit significant mucosal immune responses.

To investigate the spike-specific T cell responses on day 56 after prime immunization, splenic lymphocytes were stimulated for 24 h with peptide pools (15mers with 11 aa overlap), spanning the entire SARS-CoV-2 spike protein, and IFN-γ ELISpot assay was conducted. The results (Fig. 1e) showed that groups administered at least one dose of i.m.ChAdTS-S (i.m.ChAd>i.m.ARCoV, i.m.ARCoV>i.m.ChAd, 1×i.m.ChAd, and 2×i.m.ChAd) had the most number of IFN-γ-positive spot forming units (SFUs) per 2.5 × 10^5^ splenic lymphocytes (147, 154, 135, and 187 respectively), indicating the strongest T cell immune responses. Groups administered at least one dose of i.n.ChAd (i.n.ChAd>i.m.ARCoV, i.m.ARCoV>i.n.ChAd, 1×i.n.ChAd, and 2×i.n.ChAd) displayed T cell responses of a notable magnitude (GMTs of 36, 40, 44, and 28 SFUs per 2.5 × 10^5^ splenic lymphocytes, respectively), showing no significant difference with those of the homologous ARCoV prime-boost group (2×i.m.ARCoV). The GMT of IFN-γ-positive SFUs in the 2×i.m.ARCoV group was as 2.1-fold that of 1×ARCoV group (51 and 24 SFUs per 2.5 × 10^5^ splenic lymphocytes, respectively) but had no significant differences (*p* = 0.22).

Few studies have reported the longevity of high antibody titers induced by heterologous prime-boost vaccination regimens. We detected serum spike-specific IgG, NAb, and IgA titers following ChAdTS-S and ARCoV heterologous prime-boost in mice for 112 days after the prime vaccination. In all the immunized groups, spike-specific IgG levels (Fig. 1f, Supplementary Figs. S1–S12) and VSV-based pseudovirus NAb GMTs (Fig. 1g, Supplementary Figs. S13–S24) peaked at day 28 after the prime vaccination (7 days after the boost) and were maintained until day 112. The spike-specific IgA titers of the groups i.n.ChAd>i.m.ARCoV, 1×i.n.ChAd, and 2×i.n.ChAd were high and similar on day 14 post-prime vaccination, rising to a peak on day 28 (7 days after the boost), and lasted until day 112 (Fig. 1h, Supplementary Figs. S25, S29, and S30). The spike-specific IgA titers of the group i.m.ARCoV>i.n.ChAd were as high as those of the blank groups on day 14, increased significantly to the same peak level as those of group i.n.ChAd>i.m.ARCoV on day 28, and lasted until day 112 (Fig. 1h, Supplementary Fig. S26). IgA responses of groups administered without i.n.ChAd (i.m.ChAd>i.m.ARCoV, i.m.ARCoV>i.m.ChAd, 1×i.m.ChAd, 2×i.m.ChAd, 1×i.m.ARCoV, 2×i.m.ARCoV, i.n.Blank, and i.m.Blank) remained negative throughout the 112 days after the prime vaccination (Fig. 1h, Supplementary Figs. S27, S28, S31–S36).

In our investigation, the ChAdTS-S intranasally immunized heterologous prime-boost group (i.n.ChAd>i.m.ARCoV) had not only high neutralizing ability and systemic immune responses, but also a strong mucosal immune response and a strongT cell immunity. It may be a promising method to protect the population against SARS-CoV-2 infection. First, the i.n.ChAdTS-S priming delivered antigens to the site of infection. It initiated high mucosal immunity in the respiratory tract with systemic humoral and cellular immunity, achieving rapid and comprehensive protection. Intranasal immunization is non-invasive and easy to administer. For children who are afraid of needles, this route is more acceptable. Next, the i.m.ARCoV boosting significantly increased the IgG and NAb titers to the level as high as that elicited by 2×ARCoV. When faced with supply shortages of COVID-19 vaccines, governments can distribute new vaccines immediately without worrying about setting aside a second dose of a specific vaccine to inoculate people weeks or months later.

There are certain limitations of this investigation. First, only one group of mice of a specific age and gender were used; hence, the implications of the heterologous prime-boost strategy based on age, gender, and mice type have not been discussed. Further studies are needed to evaluate these factors. Next, the neutralizing capacities against SARS-CoV-2 variants, such as B.1.1.7 (Alpha), B.1.351 (Beta) and B.1.617.2 (Delta), were not determined after the ChAdTS-S and ARCoV heterologous prime-boost. We are working to obtain pseudovirus or live virus samples of these variants to overcome this limitation.

In conclusion, our research showed that heterologous prime-boost with ChAdTS-S and ARCoV could induce a significantly strong immune response, thereby providing reference data for optimization of vaccination programs for COVID-19 vaccines.

## Supplementary information

Supplementary Materials

## Data Availability

The original datasets are available from the corresponding author upon request.
