# Supplementary material for: Heterologous prime-boost with AdC68- and mRNA-based COVID-19 vaccines elicit potent immune responses in mice
Source: Signal Transduct Target Ther. 2021 Dec 13;6:419. doi: 10.1038/s41392-021-00843-6 (PMC8666615; doi:10.1038/s41392-021-00843-6)
Supplement: Supplementary file 1 — Supplementary Materials [file 41392_2021_843_MOESM1_ESM.docx]

Supplementary Materials for

Heterologous prime-boost with AdC68- and mRNA-based COVID-19 vaccines elicit potent immune responses in mice

Wenjuan Li, Xingxing Li, Danhua Zhao, Jingjing Liu, Ling Wang, Miao Li, Xinyu Liu, Jia Li, Xiaohong Wu, Yuhua Li

Correspondence to: [liyuhua@nifdc.org.cn](mailto:liyuhua@nifdc.org.cn) or wuxiaohong@nifdc.org.cn

**This PDF file includes:**

Materials and Methods

Figures. S1 to S36, the IgG, NAb, and IgA titers between 14 to 112 days post prime vaccination

Materials and Methods

Animals and vaccines

The experiments involving animals were carried out in compliance with the Guide for the Care and Use of Laboratory Animals of the People’s Republic of China, and approved by the Committee on the Ethics of Animal Experiments of Chinese National Institutes for Food and Drug Control (permission number: zhongjiandong(fu)2021(B)038).

Female BALB/c mice aged 4 weeks (adolescence) were provided and housed by the Chinese National Institutes for Food and Drug Control. The vaccines in this study were the chimpanzee adenovirus-vectored vaccine ChAdTS-S ( 5×10^10^ VP/0.5 mL, WALWAX Biotechnology CO., LTD., Kunming, China) and the mRNA vaccine ARCoV ( 15 µg/0.5 mL, WALWAX Biotechnology CO., LTD.). Mice were randomly divided into 12 groups (N = 10) and immunized using different vaccination regimens. The overall scheme used for group design, immunization, and immunological characterization is outlined in Figure 1a. The prime vaccination day was set as day 0, and the boost was performed on day 21. In total, 5×10^9^ VP of ChAdTS-S or 6 μg of ARCoV were used for each vaccination. Blood samples from all mice were collected on days 14, 28, 35, 42, 49, 56, 84 and 112.

Detection of the spike-specific IgG and IgA titers using ELISA

The spike-specific IgG and IgA titers in the serums were determined using ELISA. Briefly, Costar ELISA plates (Corning, NY, USA) were coated overnight with 0.2 μg SARS-CoV-2 spike protein (Sino Biological, Beijing, China). The plate was blocked with PBS + 1% BSA + 0.05% Tween 20 for 1 h at 37 °C. After washing the plates six times with PBST (PBS + 0.05% Tween 20), 4-fold serially diluted serums (initial dilution factor of 30) were added to the wells. Plates were washed six times with PBST and then incubated with horseradish peroxidase (HRP)-conjugated goat anti-mouse IgG (ZSGB-BIO, Beijing, China) (diluted at 1:10,000) or HRP-conjugated goat anti-mouse IgA (Abcam, Cambridge, UK) (diluted at 1:10,000) for 1 h at 37 °C. After washing, 3,3',5,5'-tetramethylbenzidine (TMB) (Beyotime, Shanghai, China for IgG; Sigma, Missouri, USA for IgA) was used as the substrate to detect the antibody responses at 450nm and 630 nm. The endpoint of the serum antibody titer was determined as the reciprocal of the highest dilution, which was 2.1-fold higher than the optical absorbance value of the negative control.

Recombinant VSV-based pseudovirus neutralization assay

The recombinant VSV-based SARS-CoV-2 pseudotyped virus was provided by the Division of HIV/AIDS and Sex-Transmitted Virus Vaccines, the National Institutes for Food and Drug Control. The method has been described previously. Briefly, serum samples from mice were inactivated in a water bath at 56 ℃ for 30 min. 3-fold serially diluted serum (initial dilution factor of 30) and 650 TCID50 (50% tissue culture infectious doses) of the SARS-CoV-2 pseudovirus were mixed and incubated at 37 ℃ for 1 h. Vero cells (2×10^5^ cells) were added and incubated at 37°C with 5% CO_2_ for 24 h. and the amount of pseudotyped virus entering the target cells is calculated by detecting the expression of luciferase, to obtain the neutralizing antibody content of the sample. The relative luciferase activity was measured using a luciferase assay system (Perkin-Elmer, Waltham, Massachusetts, USA). The cell control (CC) with only cells and the virus control (VC) with virus and cells are set up in each plate. The half maximal effective concentration (EC50) is calculated for the tested samples. Neutralizing antibody titer less than 30 is marked as 30 when plotting figures.

IFN-γ ELISpot assay

Splenic lymphocyte were isolated using a lymphocyte separation medium (Dakewe, Beijing, China). The isolated cells were then suspended in serum-free medium (Dakewe) for subsequent experiments. The IFN-γ positive cells were measured using a mouse IFN-γ ELISpot plus kit (Mabtech, Stockholm, Sweden). Briefly, 96-well polyvinylidene fluoride plates were washed four times with 200 µL of PBS and then blocked with RPMI-1640 medium containing 10% FBS for at least 2 h at room temperature. Freshly isolated 2.5×10^5^ Splenic lymphocyte were transferred to the plates and stimulated at 37 °C for 24 h with a peptide pool (1ug/ml per peptide, Genscript, Nanjing, China) derived from a peptide scan (15mers with 11 aa overlap) through the entire spike glycoprotein of SARS-CoV-2. Subsequently, the plates were incubated with anti-mouse IFN-γ detection antibody at room temperature for 2 h, and then with streptavidin-HRP (diluted at 1:1,000) for 1 h. After washing, 100 μL/well of a TMB substrate solution was added and developed for 10 min until distinct spots emerged. The IFN-γ-secreting cell spots were imaged and counted using ImmunoSpot S6Universal (CTL, NY, USA).

Statistical analysis

All graphs and statistical analyses were performed using GraphPad Prism v9 (GraphPad Software, San Diego, USA). One-way analysis of variance was used to determine the statistical significance among the different groups. All data were log transformed before analysis.

Figure. S1.

Temporal changes of the serum spike-specific binding IgG titers in group 1 (i.n.ChAd > i.m.ARCoV). Bars represent the geometric means with geometric SDs.

Figure. S2.

Temporal changes of the serum spike-specific binding IgG titers in group 2 (i.m.ARCoV > i.n.ChAd). Bars represent the geometric means ± SEM.

Figure. S3.

Temporal changes of the serum spike-specific binding IgG titers in group 3 (i.m.ChAd > i.m.ARCoV). Bars represent the geometric means with geometric SDs.

Figure. S4.

Temporal changes of the serum spike-specific binding IgG titers in group 4 (i.m.ARCoV > i.m.ChAd). Bars represent the geometric means with geometric SDs.

Figure. S5.

Temporal changes of the serum spike-specific binding IgG titers in group 5 (1×i.n.ChAd). Bars represent the geometric means with geometric SDs.

Figure. S6.

Temporal changes of the serum spike-specific binding IgG titers in group 6 (2×i.n.ChAd). Bars represent the geometric means with geometric SDs.

Figure. S7.

Temporal changes of the serum spike-specific binding IgG titers in group 7 (1×i.m.ChAd). Bars represent the geometric means with geometric SDs.

Figure. S8.

Temporal changes of the serum spike-specific binding IgG titers in group 8 (2×i.m.ChAd). Bars represent the geometric means with geometric SDs.

Figure. S9.

Temporal changes of the serum spike-specific binding IgG titers in group 9 (1×i.m.ARCoV). Bars represent the geometric means with geometric SDs.

Figure. S10.

Temporal changes of the serum spike-specific binding IgG titers in group 10 (2×i.m.ARCoV). Bars represent the geometric means with geometric SDs.

Figure. S11.

Temporal changes of the serum spike-specific binding IgG titers in group 11 (i.n.Blank). Bars represent the geometric means with geometric SDs.

Figure. S12.

Temporal changes of the serum spike-specific binding IgG titers in group 12 (i.m.Blank). Bars represent the geometric means with geometric SDs.

Figure. S13.

Temporal changes of the serum NAb titers to pseudovirus in group 1 (i.n.ChAd > i.m.ARCoV). Bars represent the geometric means with geometric SDs.

Figure. S14.

Temporal changes of the serum NAb titers to pseudovirus in group 2 (i.m.ARCoV > i.n.ChAd). Bars represent the geometric means with geometric SDs.

Figure. S15.

Temporal changes of the serum NAb titers to pseudovirus in group 3 (i.m.ChAd > i.m.ARCoV). Bars represent the geometric means with geometric SDs.

Figure. S16.

Temporal changes of the serum NAb titers to pseudovirus in group 4 (i.m.ARCoV > i.m.ChAd). Bars represent the geometric means with geometric SDs.

Figure. S17.

Temporal changes of the serum NAb titers to pseudovirus in group 5 (1×i.n.ChAd). Bars represent the geometric means with geometric SDs.

Figure. S18.

Temporal changes of the serum NAb titers to pseudovirus in group 6 (2×i.n.ChAd). Bars represent the geometric means with geometric SDs.

Figure. S19.

Temporal changes of the serum NAb titers to pseudovirus in group 7 (1×i.m.ChAd). Bars represent the geometric means with geometric SDs.

Figure. S20.

Temporal changes of the serum NAb titers to pseudovirus in group 8 (2×i.m.ChAd). Bars represent the geometric means with geometric SDs.

Figure. S21.

Temporal changes of the serum NAb titers to pseudovirus in group 9 (1×i.m.ARCoV). Bars represent the geometric means with geometric SDs.

Figure. S22.

Temporal changes of the serum NAb titers to pseudovirus in group 10 (2×i.m.ARCoV). Bars represent the geometric means with geometric SDs.

Figure. S23.

Temporal changes of the serum NAb titers to pseudovirus in group 11 (i.n.Blank). Bars represent the geometric means with geometric SDs. Neutralizing antibody titers less than 30 were marked as 30 when plotting figures.

Figure. S24.

Temporal changes of the serum NAb titers to pseudovirus in group 12 (i.m.Blank). Bars represent the geometric means with geometric SDs. Neutralizing antibody titers less than 30 were marked as 30 when plotting figures.

Figure. S25.

Temporal changes of the serum spike-specific binding IgA titers in group 1 (i.n.ChAd > i.m.ARCoV). Bars represent the geometric means with geometric SDs.

Figure. S26.

Temporal changes of the serum spike-specific binding IgG titers in group 2 (i.m.ARCoV > i.n.ChAd). Bars represent the geometric means with geometric SDs.

Figure. S27.

Temporal changes of the serum spike-specific binding IgA titers in group 3 (i.m.ChAd > i.m.ARCoV). Bars represent the geometric means with geometric SDs.

Figure. S28.

Temporal changes of the serum spike-specific binding IgA titers in group 4 (i.m.ARCoV > i.m.ChAd). Bars represent the geometric means with geometric SDs.

Figure. S29.

Temporal changes of the serum spike-specific binding IgA titers in group 5 (1×i.n.ChAd). Bars represent the geometric means with geometric SDs.

Figure. S30.

Temporal changes of the serum spike-specific binding IgA titers in group 6 (2×i.n.ChAd). Bars represent the geometric means with geometric SDs.

Figure. S31.

Temporal changes of the serum spike-specific binding IgA titers in group 7 (1×i.m.ChAd). Bars represent the geometric means with geometric SDs.

Figure. S32.

Temporal changes of the serum spike-specific binding IgA titers in group 8 (2×i.m.ChAd). Bars represent the geometric means with geometric SDs.

Figure. S33.

Temporal changes of the serum spike-specific binding IgA titers in group 9 (1×i.m.ARCoV). Bars represent the geometric means with geometric SDs.

Figure. S34.

Temporal changes of the serum spike-specific binding IgA titers in group 10 (2×i.m.ARCoV). Bars represent the geometric means with geometric SDs.

Figure. S35.

Temporal changes of the serum spike-specific binding IgA titers in group 11 (i.n.Blank). Bars represent the geometric means with geometric SDs.

Figure. S36.

Temporal changes of the serum spike-specific binding IgA titers in group 12 (i.m.Blank). Bars represent the geometric means with geometric SDs.
